# Perioperative Immune Checkpoint Blockade for Muscle-Invasive and Metastatic Bladder Cancer

**DOI:** 10.33696/cancerimmunol.6.081

**Published:** 2024

**Authors:** Chethan Ramamurthy, Karen M. Wheeler, Shaun Trecarten, Zaineb Hassouneh, Niannian Ji, Yifen Lee, Robert S. Svatek, Neelam Mukherjee

**Affiliations:** 1 Department of Urology, University of Texas Health San Antonio (UTHSA), San Antonio, TX 78229, USA; 2 Department of Urology and Pathology and Laboratory Medicine, University of Rochester Medical Center, Rochester, NY, USA

**Keywords:** αPD-L1 therapy, Bladder cancer, Immune checkpoint inhibitors, Immunotherapy, T cells

## Abstract

Checkpoint inhibitors offer promise in treating muscle-invasive and metastatic bladder cancer, but the optimal timing of their administration—neoadjuvant or adjuvant—remains unclear. To determine the efficacy of combining checkpoint inhibition with standard cisplatin-based chemotherapy, we conducted a phase II trial of neoadjuvant anti-PD-1 (αPD-1) and anti-CTLA-4 (αCTLA-4), in combination with cisplatin-gemcitabine, for patients with muscle-invasive bladder cancer prior to radical cystectomy. In addition, a novel murine model of spontaneous metastatic bladder cancer was used to compare the efficacy of neoadjuvant versus adjuvant anti-PD-L1 (αPD-L1) treatment. The clinical trial was closed prematurely due to the industry’s withdrawal of drug provision. Adverse events were observed in all patients; however, serious adverse events were not observed in any patient. A complete pathologic response was observed in 50% of the 4 patients enrolled. Response to treatment was significantly associated with elevated urinary T cells including CD8^+^ and IFNγ^+^ CD4^+^ T cells, suggesting potential reinforcement of immune responses by neoadjuvant αPD-1 and αCTLA-4 against bladder tumor cells. These findings suggest that combining chemotherapy and immunotherapy in the neoadjuvant setting could be safe. However, the complete response rate of this four-drug regimen was modest and emphasizes the need for randomized controlled trials to properly assess immunotherapy efficacy in the neoadjuvant setting. In corresponding murine studies, the MB49-met model consistently displayed widespread metastasis, including tumor growth in the lungs, liver, and bowel mesentery, within 20 days of subcutaneous transplantation. Mice receiving surgery plus neoadjuvant αPD-L1 or adjuvant αPD-L1 exhibited improved survival compared to those receiving only αPD-L1. However, no significant difference in survival was observed between the neoadjuvant and adjuvant αPD-L1 cohorts. Furthermore, the timing of neoadjuvant therapy administration (early vs. late) did not significantly impact survival. This study highlights the potential of perioperative immunotherapy in the treatment of locally advanced and metastatic bladder cancer.

## Introduction

Bladder cancer (BC) continues to pose a significant threat among genitourinary cancers, with high mortality rates [1]. While treatments differ based on disease stage, radical cystectomy remains the preferred option for individuals diagnosed with non-metastatic muscle-invasive BC (MIBC). Unfortunately, approximately half of the patients who undergo cystectomy face the challenge of disease relapse [2], primarily attributed to the presence of micro-metastatic disease at the time of cystectomy.

The FDA approval of immune checkpoint inhibitor (ICI) antibodies (e.g., αCTLA-4, αPD-L1, and αPD-1) and the first antibody-drug conjugate (enfortumab vedotin) has brought a transformative shift in the treatment of locally advanced and metastatic BC [3–6]. Due to the pivotal role played by the PD-1/PD-L1 and CTLA-4 immune checkpoint pathways in suppressing the immune response against cancer cells, the use of inhibitors to block these pathways has shown remarkable efficacy in managing various advanced and metastatic malignancies. Consequently, clinical trials are currently investigating the utilization of αCTLA-4 and αPD-L1/αPD-1 therapies at earlier stages of BC. This approach, known as perioperative therapy, holds particular appeal for patients with BC due to the high rates of relapse following surgical intervention. Recent findings have shown promising outcomes of checkpoint inhibitors in various cancers when administered as neoadjuvant therapies [7–9].

In adjuvant trials, treatment is initiated promptly (within 60 days) after cystectomy to enhance the immune response against cancer and eliminate any residual disease that might persist. Despite the established effectiveness of αPD-L1/αPD-1 immunotherapy in metastatic disease and its potential in the perioperative setting, our published data reveal that the antitumor efficacy of post-operative αPD-L1 therapy is compromised by the surgical procedure itself [2,10]. However, postoperative metastasis of bladder cancer and diminished immune responses of T cells against bladder tumors as a result of invasive interventions suggest that surgery can have detrimental effects on outcomes in bladder cancer and potentially diminish the response to adjuvant immunotherapy.

This study assesses the impact of perioperative immunotherapy in BC and aims to provide insights into treatment implications in BC management, specifically examining neoadjuvant immunotherapy with cisplatin-gemcitabine in patients with MIBC and also comparing it to adjuvant immunotherapy using a preclinical metastatic BC model.

## Methods

### Trial design

#### Design and participants:

This Phase II trial aims to determine safe and tolerable doses of αCTLA-4 and αPD-1 in combination with neoadjuvant cisplatin and gemcitabine. As a secondary objective, the study will evaluate the clinical benefits of adding neoadjuvant αCTLA-4 and αPD-L1 concurrently with cisplatin and gemcitabine neoadjuvant chemotherapy in patients with muscle-invasive, non-metastatic urothelial BC (cT2-4N0-1M0) eligible for cisplatin-based neoadjuvant chemotherapy prior to radical cystectomy. The study (NCT04430036) was approved by the local institutional regulatory board (HSC20200027H) and publicly registered under CTMS# 19–0193. Written informed consent was obtained from all participating patients, and the study adheres to the principles of the Declaration of Helsinki. Blood and urine samples were collected for flow cytometry or immunohistochemistry (IHC). Eligibility criteria include muscle-invasive, non-metastatic urothelial carcinoma of the bladder (cT2-4, N0-1, M0), Eastern Cooperative Oncology Group performance status of 0–1, creatinine clearance (CrCl) ≥ 50 mL/min, hearing loss ≤ grade 2, peripheral neuropathy ≤ grade 2, New York Heart Association class <III congestive heart failure, and eligibility to receive gemcitabine. Additional criteria encompass absolute neutrophil count >2,000/mcL, hemoglobin >9.0 mg/mL, platelets >100,000/mcL, normal or known elevated total bilirubin with normal conjugated bilirubin level, AST/ALT <3X institutional normal limits, CrCl >50 mL/min/1.73m^2^, estimated by CKD-EPI or measured with a 24-hour urine collection (whichever is greater), and age ≥ 18 years. Exclusions comprise prior receipt of checkpoint inhibitors (αPD-1, αPD-L1, or αCTLA-4 antibodies) or anticancer medications, persisting toxicity of National Cancer Institute Common Terminology Criteria for Adverse Events version 5.0 (NCI-CTCAE) Grade >1 severity from prior therapy, severe hypersensitivity reactions to fully human monoclonal antibodies (NCI-CTCAE Version 5.0 Grade ≥3), history of anaphylaxis or uncontrolled asthma, active or history of autoimmune disease, systemic treatment with corticosteroids (>10mg daily prednisone equivalents) or other immunosuppressive medications within 14 days before the first dose of study drug, uncontrolled intercurrent illnesses including infection, interstitial lung disease, or active non-infectious pneumonitis, symptomatic congestive heart failure, unstable angina pectoris, uncontrolled cardiac arrhythmia, social situations limiting compliance with study requirements, intolerance or allergic reactions to compounds similar to αPD-L1 and αCTLA-4, pregnancy or breastfeeding, and receipt of a live vaccine within 30 days prior to the first dose of study drug.

#### Treatment:

The treatment regimen consists of AGEN1884 (αCTLA-4 human monoclonal antibody) at a dosage of 1 mg/kg every 6 weeks, along with AGEN2034 (αPD-1 human monoclonal antibody) at a dosage of 300 mg every 3 weeks. This is administered concurrently with cisplatin at a dosage of 70 mg/m^2^ on day 1 and gemcitabine at a dosage of 1000 mg/m^2^ on days 1 and 8 of a 21-day cycle, for a maximum of 4 cycles. AGEN1884 and AGEN2034 are administered on day 1 of cycles 1 and 3, while AGEN2034 alone is administered on day 1 of cycle 2, following cisplatin and gemcitabine. When both AGEN1884 and AGEN2034 are given on the same day, AGEN1884 is administered prior to AGEN2034.

#### Safety:

Safety was evaluated during each visit through the assessment of treatment-emergent adverse events according to the National Cancer Institute Common Terminology Criteria for Adverse Events version 5.0, as well as physical examinations, vital signs monitoring, weight measurements, clinical laboratory tests, and ECOG performance status. The monitoring and documentation of adverse events commenced with the initiation of the initial study treatment.

#### Immune biomarker analysis by flow cytometry:

Peripheral blood was obtained through peripheral venipuncture into lithium heparin vials (BD, #367880) and urine samples were collected at baseline, as well as at select time points (C3D1, cystectomy, and 3-month follow-up). Peripheral blood mononuclear cells (PBMCs) were extracted from patient blood samples using Ficoll-Paque gradients (GE Healthcare). These PBMCs were suspended in a freezing medium (complete Roswell Park Memorial Institute (RPMI) with 50% fetal bovine serum and 10% dimethyl sulfoxide (DMSO) (Corning, Fisher Scientific) and then cryopreserved at −150°C until further analysis.

The urine sample processing involved transferring the entire urine content into a 50 mL conical tube and centrifuging it at approximately 500 g/rcf for 5 minutes at 4°C. Subsequently, the supernatant was carefully decanted or transferred to another conical tube for analysis, and 1 mL portions were aliquoted into 3–5 vials for storage at −80°C. The remaining urine cell pellet was then resuspended in serum-free RPMI, and a 500 μL sample was taken for cell count. After repeat centrifugation, the supernatant was discarded, and the urine pellet was broken up again. Based on cell count results, the urine pellet was resuspended in cR-10 freezing media containing 20% DMSO. The final mixture was aliquoted into cryogenic vials on ice, transferred to a CoolCell container, and placed in a −80°C freezer, with subsequent transfer to a −150°C freezer after 24 hours until further analysis.

PBMCs and urinary cells were thawed in complete RPMI and counted with a Vi-cell XR (Beckman Coulter) before resuspension in flow buffer (2% fetal bovine serum in PBS). A total of 1×10^6^ cells per sample were stained and analyzed using an LSR II flow cytometer and FACSDiva software (BD Bioscience, v6), as described previously. Validated commercial reagents, including LIVE/DEAD Fixable Aqua Dead Cell Stain Kit (Life Technologies), fluorochrome-conjugated monoclonal antibodies against CD45 (clone HI30), CD3 (clone HIT3a), CD4 (clone OKT4), CD8 (clone SK1), and IFNγ (clone 4S.B3). For IFNγ staining, cells were stimulated with a Leukocyte Activation Cocktail with GolgiPlug (BD Biosciences #550583) for 5 hours following the manufacturer’s protocol. Subsequently, they were surface stained, fixed, permeabilized with fixation and permeabilization solution (BD Biosciences, #554722) according to the manufacturer’s protocol, and then stained for intracellular cytokines.

### Murine experiments

#### Cell culture:

The MB49 spontaneous metastatic cell line (gift from Dr. Yifen Lee) is a carcinogen-derived urothelial cell carcinoma originally derived from C57BL/6 male mice [7,11]. The MB49-met cells were cultured in complete RPMI media supplemented with 10% fetal bovine serum, 0.1% penicillin/streptomycin, and 1% fresh L-Glutamine for maintenance purposes.

#### Animals:

All animal experiments were conducted in compliance with the guidelines established by the local animal ethics committee and were approved under the Institutional Animal Care and Use Committee (IACUC) protocol (20120040AR). C57BL/6 male mice, aged 6–8 weeks, were either obtained from Jackson Laboratory or bred in-house under conventional housing conditions.

#### Tumor inoculation:

MB49-met cells were cultured for at least one passage until reaching approximately 70% confluency. Subsequently, the cells were harvested and subcutaneously injected (s.c.) into the mice at a concentration of 1×10^6^ cells in 100 μL of PBS per flank/tumor site. Tumor measurements were taken using calipers every three days, and the tumor volume was calculated using the formula (length x width x width)/2 (mm^3^).

#### Surgical tumor excision:

For the excision of subcutaneous tumors, mice were anesthetized with a mixture of ketamine (80 mg/kg), xylazine (8 mg/kg), and Accepromazin (1 mg/kg) injected at a dose of 300 μL/30 g of body weight. The surgical procedure was conducted under sterile conditions and in a negative pressure hood, with the mice placed on a heating pad. The mice were first shaved, and the skin was prepared with betadine. Subsequently, the tumors and overlying skin were sharply removed, and the skin was reapproximated using a 4–0 Vicryl suture.

#### Treatment:

For neoadjuvant and adjuvant immunotherapy, αPD-L1 (BioXcell, clone - 10F.9G2) and isotype rat IgG (BioXcell) were utilized. Intraperitoneal injections of 100 μg doses were administered every 3 days for a total of 3 doses. On day 14 post-tumor inoculation, mice were assigned to receive neoadjuvant αPD-L1, neoadjuvant isotype rat IgG, or undergo primary tumor removal (adjuvant). In the neoadjuvant groups, αPD-L1 or isotype control was given on days 14, 17, and 20. Surgery for the neoadjuvant-treated group was performed on day 24. In the adjuvant groups, αPD-L1 or isotype rat IgG was given on days 19 (5 days post-operatively), 22, and 25. The study also examined the effects of early neoadjuvant therapy (antibody administered on days 10, 13, and 17; surgery on day 21) and late adjuvant therapy (surgery on day 21; antibody administered on days 24, 27, and 30).

#### Statistics:

Subcutaneous tumor growth curves between groups were compared with the 2-way analysis of variance (ANOVA). Group differences in the number and percentage of immune cells, mean fluorescence intensity and cytokine production were assessed using unpaired t-tests. Survival curves were evaluated using the Log-rank test. Two-sided p-values were calculated and p <0.05 was considered statistically significant. GraphPad Prism 5–6 or Stata IC/10.1 were employed for conducting the statistical analyses.

## Results

### Study population and adverse events

#### Study population:

Prior to premature study closure (unexpected withdrawal of support by Agenus Inc.), 4 patients (2 men, and 2 women) were included (from 10/2020 to 10/2021), with a median age of 58.5 [IQR 49.5, 67.75]. All four patients are white, one of whom is of Spanish/Hispanic/Latino origin. The median Charlson comorbidity index (CCI) is 3.5 [IQR 2.5, 4.5]. Baseline ECOG was 0 in three patients and 1 in one patient. End of the study ECOG scores were all similar apart from one patient with an increase from 0 to 1. Clinical T staging at study entry was cT2 for three patients, and unknown in one. None of the patients had metastatic disease on imaging at study entry. All patients received 4 cycles of neoadjuvant therapy and proceeded with cystectomy.

#### Adverse events (AEs):

**Figure 1.**
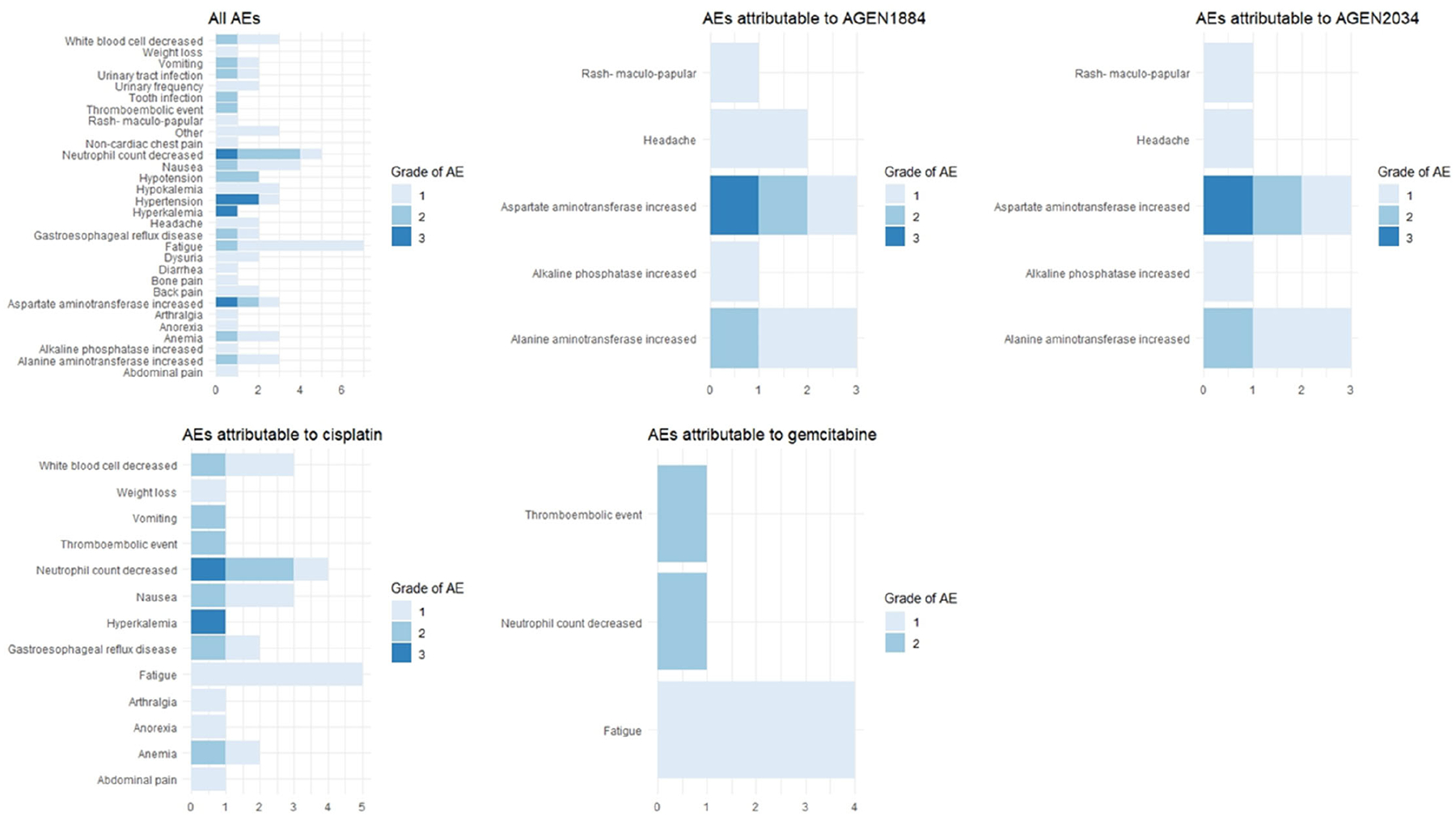
Safety Assessment of the Trial Drugs. The total adverse events (AEs) and medication-attributable AEs are categorized by grade, providing a comprehensive overview of the safety profile for each medication in the trial.

Overall, 65 AEs were reported (Table 1), of which the most common were fatigue (N=7, 10.8%), reduced neutrophil count (N=5, 7.7%), and nausea (N=4, 6.2%). Of these AEs, 5 (7.7%) were grade 3, 16 (24.6%) were grade 2 and 44 (67.7%) were grade 1. There were no grade 4 AEs. Attributable grade 3 AEs were observed with one patient having decreased neutrophil count (probably due to cisplatin), and one patient having both elevated AST (probably due to AGEN1884 or AGEN2034) and hyperkalemia (possibly due to cisplatin). All attributable AEs are also listed (Figure 1).

#### Postoperative course and outcomes:

Postoperative complications occurred in 2 patients. One patient developed Clavian-Dindo [CD] Grade 1 ileus and the other developed sepsis (CD Grade 2). Median hospital length of stay was 6 days (IQR 5.5, 8). Pathologic complete response (pCR) was observed in 2 patients who achieved ypT0 on pathology. Pathologic downstaging occurred in 3 patients, two with pCR and one who was downstaged to ypT1. One patient remained ypT2a. All patients were ypN0 (median nodes resected 22 [IQR 20.75, 22.5]). No meaningful follow-up information is available due to the closure of the study.

### Urinary T cell subsets are significantly increased in patients who showed therapeutic response compared to non-responding patients

**Figure 2.**
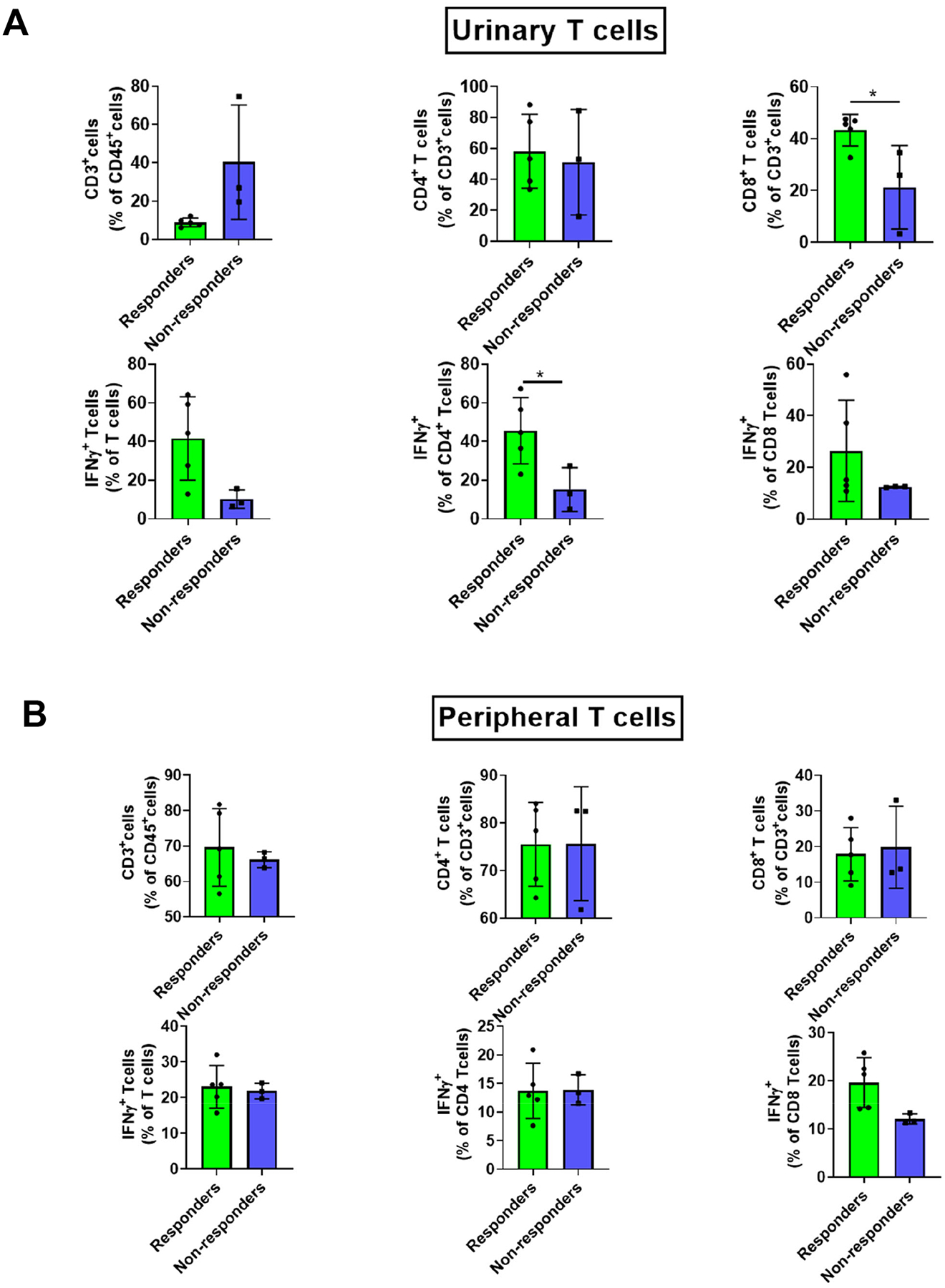
Neoadjuvant anti-PD-L1 in combination with cisplatin-gemcitabine increases the frequency of urinary CD8^+^ and IFNγ^+^ CD4^+^ T cells compared with their PBMC counterparts in patients with muscle-invasive bladder cancer. Urinary T cells **(A)** and PBMC-derived T cells **(B)** were analyzed; phenotypes were determined using flow cytometry and compared between responders and non-responders. P: unpaired, two-tailed t-tests. P value threshold for significance <0.05.

Perioperative immune checkpoint inhibitors (ICIs) have demonstrated therapeutic advantages in various cancers by enhancing the frequency and functionality of T cells [12,13]. In this study, we investigated the impact of perioperative α-CTLA-4 combined with αPD-1 on urinary and peripheral T cells in treatment-responsive individuals versus non-responsive patients. In assessing T cell alterations among responders, we investigated changes in T cell populations throughout the treatment course. Significantly elevated levels of urinary CD8^+^ and IFNγ CD4^+^ T cells were noted in responders, contrasting with non-responders (Figure 2A). No substantial changes were detected in peripheral T cells for both responsive and non-responsive individuals (Figure 2B). These findings imply distinct effects of perioperative ICIs on tumor-infiltrating T cells, as evidenced by variations in the frequency and cytokine secretion profiles of urinary T cell subsets, commonly used as proxies of the tumor immune landscape [14,15] in individuals who successfully responded to the perioperative treatments.

### Development of a novel spontaneous preclinical model of metastatic bladder cancer

Following the subcutaneous inoculation of MB49-met in B6 WT mice, evident pulmonary metastases were observed between days 14 and 17 (Supplementary Figure 1A and 1B). The growth rate of the primary subcutaneous MB49-met tumor was slower compared to the wild-type MB49, requiring a higher initial tumor cell count of 1×10^6^ cells (compared to 0.2×10^6^ cells for wild-type MB49) for tumor uptake (Supplementary Figure 1A and 1B). According to STR profiling performed by ATCC and CLASTR similarity search on the Cellosaurus database, MB49-met demonstrated an approximate 85% match to MB49.

Subcutaneous tumor growth in the MB49-met model reached 1000 mm^3^ around day 20, necessitating sacrifice due to the excessive tumor burden. However, the surgical removal of the primary tumor on day 20 significantly improved survival, with 50% of mice surviving until day 41. Gross examination revealed that subcutaneous MB49-met tumors metastasized to various sites, including the lungs, liver, kidneys, mesentery, and soft tissues of the neck and retroperitoneum (Supplementary Figure 1C). Despite the surgery, the overall survival rate in this model approached 100% mortality by day 55 (Supplementary Figure 1D).

### Neoadjuvant αPD-L1 and adjuvant αPD-L1 improved survival compared with αPD-L1 therapy alone

**Figure 3.**
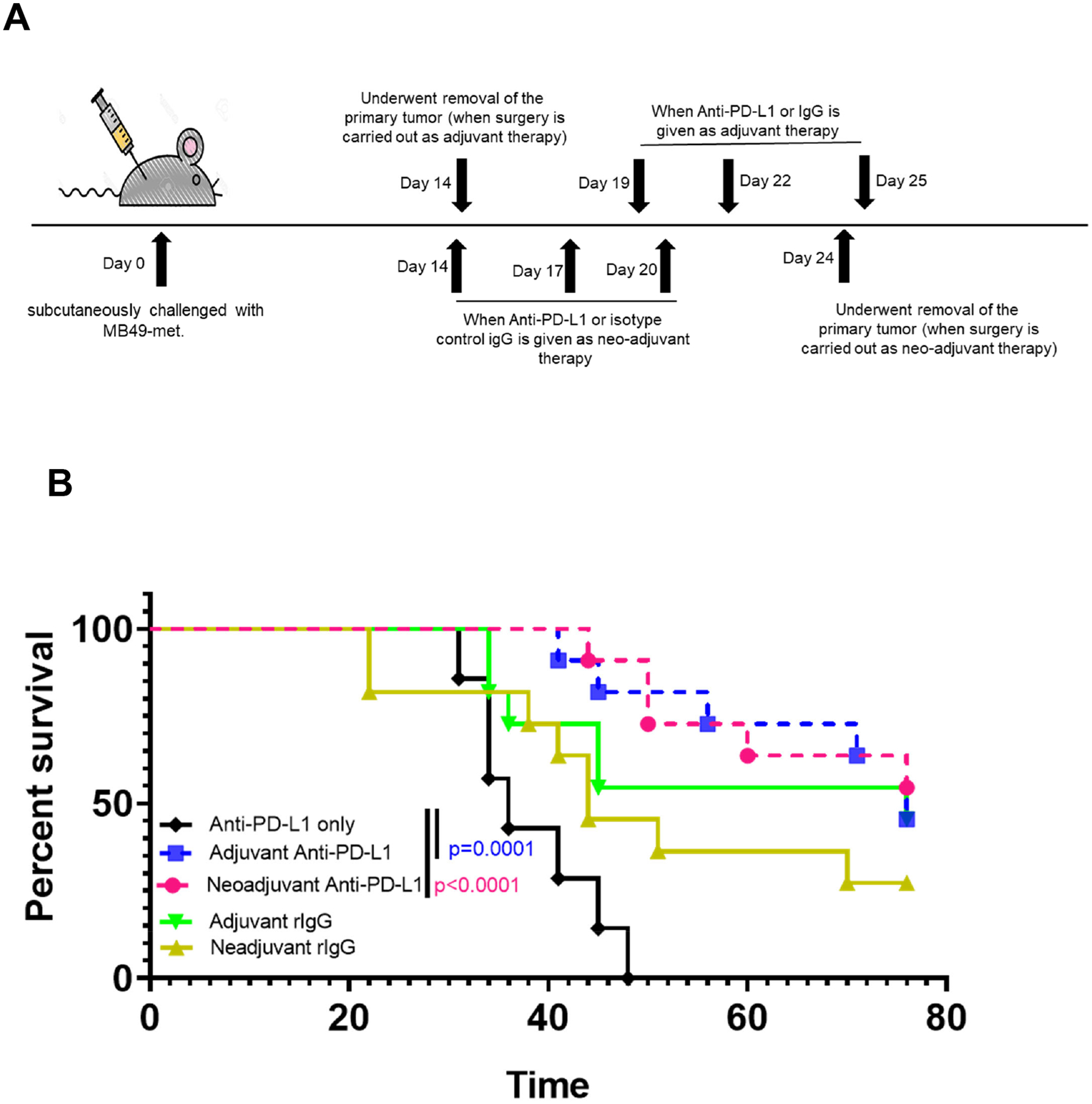
Neoadjuvant anti-PD-L1 plus surgery and adjuvant anti-PD-L1 plus surgery were improved over anti-PD-L1 therapy alone. (**A**) Timeline of immune therapy in bladder cancer treatment: On day 14 post tumor inoculation with MB49-met cells subcutaneously, mice were given anti-PD-L1 (neoadjuvant), isotype rIgG (neoadjuvant), or underwent removal of the primary tumor (adjuvant). Neoadjuvant anti-PD-L1 or isotype control was given on days 14, 17, and 20. Surgery for the neoadjuvant-treated group was performed on day 24. Adjuvant anti-PD-L1 or isotype rIgG was given on days 19 (5 days post-operatively), 22, and 25 (**B**) Survival is followed in the different treatment groups as mentioned in (**A**).

To assess the efficacy of neoadjuvant versus adjuvant immunotherapy, mice were treated with αPD-L1 or isotype control antibody either before (neoadjuvant) or after (adjuvant) surgical excision of the subcutaneous tumor (as depicted in Figure 3A). Both the neoadjuvant and adjuvant treatment groups exhibited a significant improvement in survival compared to mice treated with αPD-L1 alone (p<0.0001 and p=0.0001, respectively). Mice receiving neoadjuvant treatment followed by surgery and αPD-L1 displayed a trend towards enhanced survival when compared to the rIgG + surgery control group (p=0.08) (Figure 3B). Furthermore, no significant disparity in survival was noted between mice treated with neoadjuvant versus adjuvant anti-PD-L1 (Figure 3B).

### Timing of surgery and neoadjuvant therapy do not affect survival

**Figure 4.**
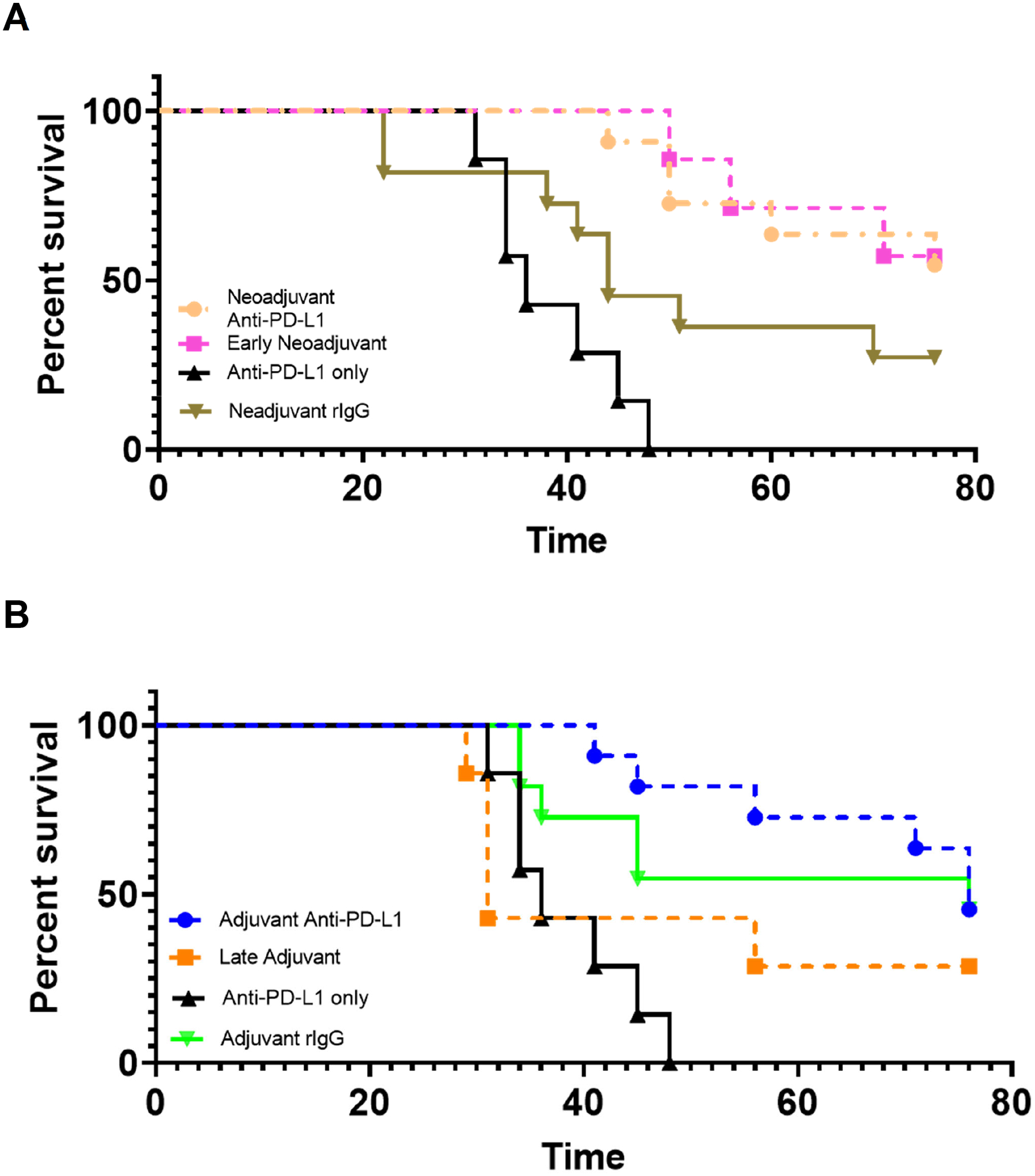
Timing of surgery and neo-adjuvant therapy do not affect survival. (**A**) On day 14 post-tumor inoculation with MB49-met cells subcutaneously, mice were given anti-PD-L1 (neoadjuvant), and neoadjuvant anti-PD-L1 was given on days 14, 17, and 20. Surgery for the neoadjuvant-treated group was performed on day 24. Early neoadjuvant therapy (antibody administered at days 10, 13, and 17; and surgery at day 21). Adjuvant anti-PD-L1 was given on day 19 (5 days post-operatively), 22, and 25 Late adjuvant therapy (surgery at day 21 and antibody administered on days 24, 27, and 30) was also explored. (**B**) Survival is followed in the different treatment groups as mentioned in (**A**).

To investigate whether the timing of surgery in relation to perioperative immunotherapy influenced survival outcomes, we established early neoadjuvant and late adjuvant groups. In the neoadjuvant group, when αPD-L1 treatment was initiated early and surgery performed on day 21, there was no significant difference in outcome (p=0.87) (Figure 4A). Similarly, in the adjuvant group, when surgery was delayed to day 21 instead of day 14, there was also no statistically significant difference in outcome (p=0.26), although there seemed to be a trend towards worsened survival (Figure 4B).

## Discussion

Radical cystectomy remains the standard treatment for patients with MIBC, however, patients often relapse, highlighting the significance of perioperative strategies [16]. BC is known to have one of the highest cancer mutational loads [17], which has been linked to favorable responses to immunotherapies in other cancer types [18]. Consequently, the rationale for using immunotherapy in BC is compelling, and FDA approvals of all 5 antibodies targeting ICIs such as CTLA-4, PD-L1, and PD-1 further support this approach in BC [19]. However, so far, there is a lack of consensus regarding the suitability of neoadjuvant or adjuvant immunotherapy in MIBC and metastatic BC [16]. Additionally, there is uncertainty about whether a single ICI or a combination of ICIs with other agents is more appropriate [16]. Our study aimed to evaluate the efficacy of combining neoadjuvant αCTLA-4 and αPD-1 with cisplatin and gemcitabine neoadjuvant chemotherapy in patients with MIBC.

The findings from this study provide evidence for the potential role of neoadjuvant αCTLA-4 and αPD-1 in enhancing both the frequency and functionality of T cells, which may lead to improved post-surgical outcomes. Notably, patients who exhibited successful responses to neoadjuvant therapy showed a significant increase in CD8^+^ T cells, along with heightened secretion of IFNγ, indicating potential T cell activation resulting from neoadjuvant αCTLA-4 and αPD-1 treatment. These effects were specifically observed in the urinary immune cells, which are often considered surrogates for the bladder tumor microenvironment. However, no significant changes were noted in peripheral immune cells among these patients. The evidence presented in this study suggests that neoadjuvant αCTLA-4 and αPD-1 treatment might augment T cell activation within the tumor, warranting further investigation into their effects on tumor-infiltrating T cells in BC.

Further, the impact of radical surgery on metastatic disease and post-operative BC therapy response remains unclear. The pro-metastatic effects of surgery were first described in 1913 [20]. Since then, several studies have shown that surgery may contribute to the development of metastatic cancer [21,22]. Complications during the post-operative period have also been associated with a worse prognosis and metastatic disease [23,24]. It has been shown that the initial surgical resection may interfere with the host immune system leading to immune suppression and the development of metastasis [25,26].

Surgery suppresses cell-mediated immunity [27], including eliciting profound effects on T cells which are key mediators of antitumor immunity [28,29]. Endogenous immunity prevents nascent malignant cell clusters from becoming clinically evident cancers through a process initially known as immune surveillance. Immune surveillance relies largely on T cells, although other immune cells and mediators participate [30,31]. Therefore, the increase in T cell frequency and function achieved by neoadjuvant αCTLA-4 and αPD-1 could be advantageous for maintaining a robust immune surveillance system, ensuring the eradication of micro-metastatic or minimal residual cancer after extirpative surgery.

The relatively short period of immune suppression during the perioperative phase is a critical factor in tumor escape and cancer metastasis. Nevertheless, these concepts remain underexplored in the context of metastatic urologic tumors. Additionally, the lack of a suitable murine model for metastatic BC that could accurately replicate the conditions of radical surgery in patients has posed challenges for evaluating neo or adjuvant immune therapy. Our newly developed metastatic model closely mimics the real-life scenario of radical cystectomy in patients, providing an opportunity to assess the efficacy of both neoadjuvant and adjuvant immune therapies. Utilizing this novel BC metastasis murine model, we investigated the survival benefits of neoadjuvant and adjuvant αPD-L1 therapy. Our results demonstrated that prompt surgery significantly improved survival, and the combination of adjuvant and neoadjuvant immunotherapy with surgery yielded superior outcomes when compared to αPD-L1 therapy alone. However, even though perioperative treatment plays a crucial role in enhancing curative outcomes, factors such as the patient’s health status, the most suitable timing for surgery, and the incorporation of predictive biomarkers play a significant role in deciding the treatment approach. Preoperative therapy has the potential to induce tumor shrinkage and support the process of tumor resection. However, it also carries the risk of delaying surgical intervention and diminishing the opportunity for achieving R0 resection. Consequently, it is vital to evaluate the surgical results associated with diverse neoadjuvant immunotherapies. Presently, numerous trials are underway, with the majority of results yet to be published. We anticipate that these trials’ findings will offer valuable insights and clarity regarding the utility of perioperative immune therapy in MIBC and metastatic BC.

There are limitations to our study. The sample size in our study was limited due to the premature closure of the trial and the effectiveness of the four-drug regimen in achieving a complete response was moderate. This highlights the necessity for randomized controlled trials to adequately evaluate the effectiveness of immunotherapy in the perioperative context. Flow cytometric analysis of immune cells was performed using urinary cells and peripheral blood mononuclear cells (PBMCs) due to the unavailability of tumor cell suspensions. Despite this limitation, urinary immune cells often mimic the phenotype of tumor-infiltrating immune cells [14,15]. We did not evaluate additional time points during the treatment cycles, but their examination could be informative. Despite utilizing subcutaneous primary implantation in our metastatic model, it remains distinctive as one of the rare models that replicate the real-life context of radical cystectomy in patients and provides a valuable chance to assess the efficacy of both neoadjuvant and adjuvant immune therapies.

In conclusion, our study thoroughly investigated the effects of perioperative immunotherapy in patients with MIBC and metastatic BC. The inclusion of αCTLA-4 and αPD-1/αPD-L1 in perioperative chemotherapy demonstrated potentially beneficial effects on immune cells, particularly in enhancing T cell function, and may lead to improved survival outcomes. These conclusions are supported by evidence from both our human data and our pre-clinical mouse BC model. Further validation and strengthening of our findings require conducting future trials with larger patient cohorts and comprehensive murine studies. These efforts will enhance our understanding of the mechanisms and efficacy of perioperative immunotherapy in BC treatment.

## Supplementary Material

JCAI-24-081-Supplementary-file

## Figures and Tables

**Table 1.** Counts(%) and grades of all reported adverse events.

Adverse Event	Count (%)	Graded	Grade 2	Grade 3
Fatigue	7 (10.8)	6	1	-
Neutrophil count decreased	5 (7.7)	1	3	1
Nausea	4 (602)	3	1	-
Anemia	3 (4.6)	2	1	-
Aspartate aminotransferase increase	3 (4.6)	1	1	1
Hypertension	3 (4.6)	1	-	2
Hypokalemia	3 (4.6)	3	-	-
White blood cell decreased	3 (4.6)	2	1	-
Alanine aminotransferase increased	3 (4.6)	2	1	-
Other	3 (4.6)	3	-	-
Urinary tract infection	2 (3.1)	1	1	-
Urinary frequency	2 (3.1)	2	-	-
Vomiting	2 (3.1)	1	1	-
Hypotension	2 (3.1)	-	2	-
Headache	2 (3.1)	2	-	-
Gastroesophageal reflux disease	2 (3.1)	1	1	-
Dysuria	2 (3.1)	2	-	-
Back pain	2 (3.1)	2	-	-
Diarrhea	1 (1.5)	1	-	-
Alkaline phosphatase increased	1 (1.5)	1	-	-
Tooth infection	1 (1.5)	-	1	-
Rash maculo-papular	1 (1.5)	1	-	-
Hyperkalemia	1 (1.5)	-	-	1
Weight loss	1 (1.5)	1	-	-
Abdominal pain	1 (1.5)	1	-	-
Arthralgia	1 (1.5)	1	-	-
Bone pain	1 (1.5)	1	-	-
Thromboembolic event	1 (1.5)	-	1	-
Non-cardiac chest pain	1 (1.5)	1	-	-
Anorexia	1 (1.5)	1	-	-
Total (%)	65	44 (67.7%)	16 (24.6%)	5 (7.7%)
