# Supplementary material for: Perioperative Immune Checkpoint Blockade for Muscle-Invasive and Metastatic Bladder Cancer
Source: J Cancer Immunol (Wilmington). Author manuscript; Available in PMC 2024 May 23. (PMC11113005; doi:10.33696/cancerimmunol.6.081)

Supplementary Figure 1. Development of MB49 metastasis model. (A-D) 1X10^{6} MB49^{met} cells were injected subcutaneously on one shaved flank of mice to generate tumors. (A) Tumors were measured using calipers every 3 days. Tumor volume was calculated using (length x width x width)/2. (B) Pathology scores of lungs were plotted across days after tumor inoculation. (C) Representative picture of lung mets in mice sacrificed on day 41 after tumor challenge. The picture shows Mets on the right hind leg and left neck, mesenteric mets, and lung mets. (D) Survival was followed over a period of ~10 weeks.

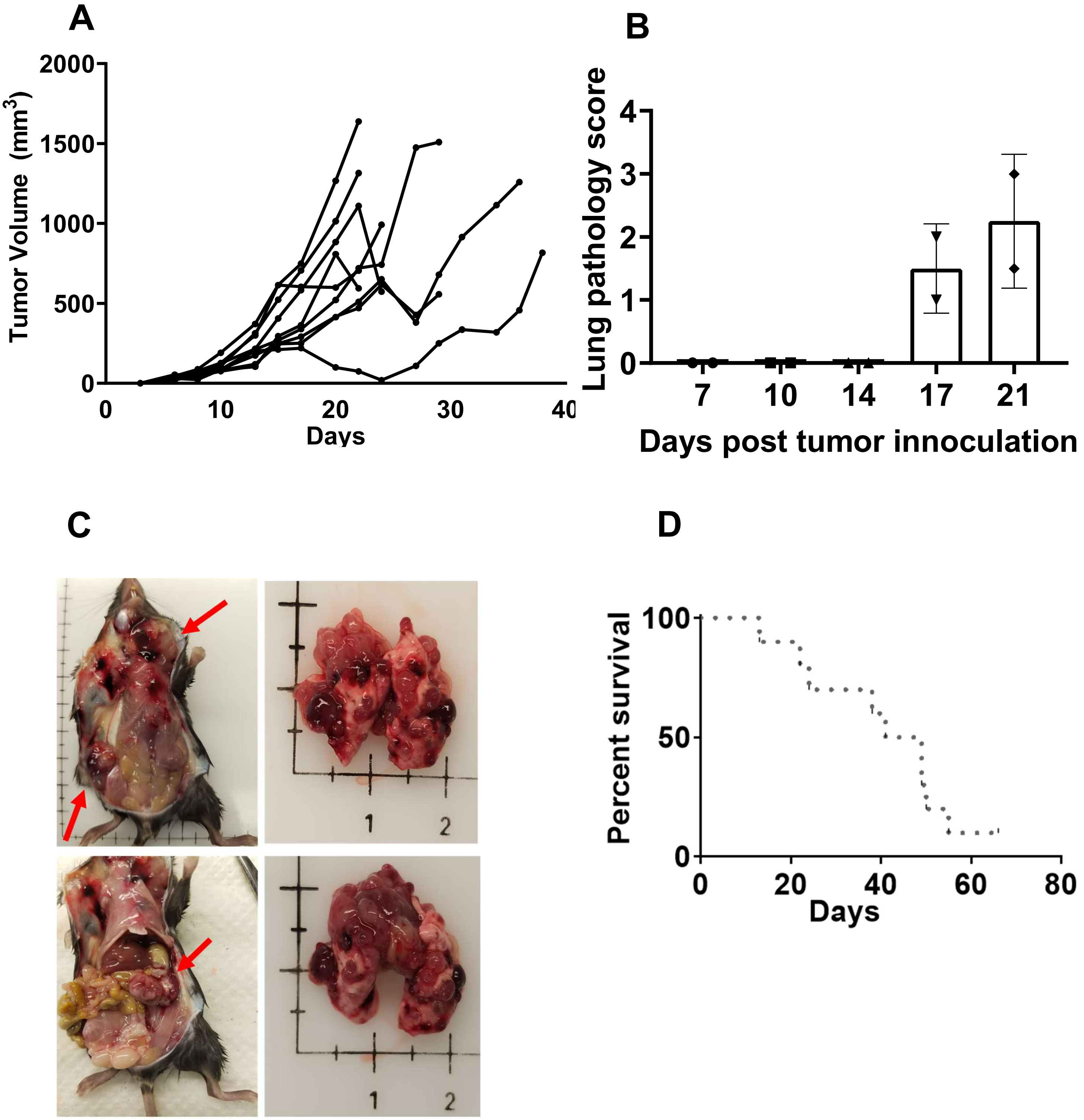

Supplement: JCAI-24-081-Supplementary-file [file NIHMS1994349-supplement-JCAI-24-081-Supplementary-file.pdf]
